# How Well the Constructs of Health Belief Model Predict Vaccination Intention: A Systematic Review on COVID-19 Primary Series and Booster Vaccines

**DOI:** 10.3390/vaccines11040816

**Published:** 2023-04-07

**Authors:** Yam B. Limbu, Rajesh K. Gautam

**Affiliations:** 1 Feliciano School of Business, Montclair State University, 1 Normal Ave., Montclair, NJ 07043, USA; 2 Department of Anthropology, Dr. Harisingh Gour Central University, Sagar 470003, MP, India; rkgautam@dhsgsu.edu.in

**Keywords:** health belief model, HBM, COVID-19, vaccination intention, primary series vaccines, boosters, systematic review

## Abstract

This systematic review synthesizes the findings of quantitative studies examining the relationships between Health Belief Model (HBM) constructs and COVID-19 vaccination intention. We searched PubMed, Medline, CINAHL, Web of Science, and Scopus using the Preferred Reporting Items for Systematic Reviews and Meta-Analyses (PRISMA) guidelines and identified 109 eligible studies. The overall vaccination intention rate was 68.19%. Perceived benefits, perceived barriers, and cues to action were the three most frequently demonstrated predictors of vaccination intention for both primary series and booster vaccines. For booster doses, the influence of susceptibility slightly increased, but the impact of severity, self-efficacy, and cues to action on vaccination intention declined. The impact of susceptibility increased, but severity’s effect declined sharply from 2020 to 2022. The influence of barriers slightly declined from 2020 to 2021, but it skyrocketed in 2022. Conversely, the role of self-efficacy dipped in 2022. Susceptibility, severity, and barriers were dominant predictors in Saudi Arabia, but self-efficacy and cues to action had weaker effects in the USA. Susceptibility and severity had a lower impact on students, especially in North America, and barriers had a lower impact on health care workers. However, cues to action and self-efficacy had a dominant influence among parents. The most prevalent modifying variables were age, gender, education, income, and occupation. The results show that HBM is useful in predicting vaccine intention.

## 1. Introduction

The outbreak of COVID-19 has affected the world severely. As of 9 March 2023, over 759 million global cases and over 6.8 million deaths have been reported [1]. The virus still poses serious health threats, especially to older adults and those with underlying comorbidities. People’s acceptability and demand for COVID-19 vaccines and their intentions to take the COVID vaccine are slowly fading away, and this trend is even worse in the case of booster doses.

Since vaccination intention is pivotal to the success of mass vaccination campaigns as well as to the attaining of herd immunity, it is essential to understand the health beliefs that influence vaccination intention against COVID-19. Some reviews have been conducted focusing on the factors associated with COVID-19 vaccination intention. These reviews analyzed COVID-19 vaccination intentions across genders [2] and healthcare workers [3], and between healthcare workers and the general adult population [4]. Two studies conducted rapid reviews, a simplified approach to systematic reviews [5,6]. Two studies performed scoping reviews to explore broad factors such as demographic, social, and contextual factors that influenced the intention to use COVID-19 vaccines [7,8]. Wang et al. [9] and Chen et al. [10] estimated the COVID-19 vaccine acceptance rate and identified predictors associated with COVID-19 vaccine acceptance. However, these studies did not focus on the health belief model (HBM) and its constructs (i.e., perceived susceptibility, perceived severity, perceived benefits, perceived barriers). To date, only one study has systematically reviewed the extant literature on HBM [11], but it focused on vaccine hesitancy. In conclusion, prior systematic reviews have focused on narrow topics and rapid and scoping reviews. As of yet, no systematic review has addressed HBM’s utility in predicting COVID-19 vaccination intention.

Hence, the purpose of the current systematic review was to analyze the research that used the HBM as a theoretical framework for understanding vaccination intention against COVID-19. We reported the prevalence of HBM constructs influencing COVID-19 vaccination intention. These results were further broken down by vaccine type (primary series versus booster doses), data collection year, country, continent, and sample type. In addition, we provided an up-to-date and comprehensive review of the literature by including articles published during 2020–2023, and those studies covering booster/third dose and parents’ or caregivers’ vaccination intention to vaccinate their young children for COVID-19. Finally, we reported the prevalence of HBM modifying variables, including demographic variables (e.g., age, gender, race, ethnicity, education, income, marital status) and structural variables (e.g., knowledge about a given disease, prior contact with the disease) that were significantly associated with COVID-19 vaccination intention. 

## 2. Methodology

This systematic review was carried out in accordance with the guidelines of the Preferred Reporting Items for Systematic Review and Meta-Analysis (PRISMA) [12,13]. The ROBIS (Risk of Bias in Systematic Reviews) tool [14] was used to evaluate the quality of included studies and the risk of bias. 

### 2.1. Eligibility

#### 2.1.1. Inclusion Criteria

We included quantitative studies that used the HBM framework and statistical methods to examine associations between HBM constructs and COVID-19 vaccination intention for both primary series and booster doses. To ensure the quality of scientific investigation, we included only studies published in peer-reviewed journals. Other inclusion criteria were articles published in English between December 2019 and February 2023.

#### 2.1.2. Exclusion Criteria

We excluded (1) studies that reported only vaccination intention against COVID-19 without applying HBM constructs; (2) studies that reported vaccination intention against COVID-19 with HBM constructs but which did not perform a quantitative analysis; (3) qualitative studies, non-peer reviewed studies, and conference proceedings; (4) reviews, comments, case reports, editorials and letters; and (5) grey literature.

### 2.2. Search Strategy

A comprehensive search for published literature was conducted in the selected databases: PubMed, Web of Science, CINAHL, and Scopus using various key words such as “health belief model” or “HBM”, “vaccination intention” or “vaccine acceptance”, “COVID-19” or “coronavirus” or “SARS-CoV-2”, “first or second dose” or “primary series”, “booster shot or dose” or “third dose”.

The search was conducted from 1 January 2022 to 28 February 2023. Full length papers published between December 2019 and February 2023 were retrieved for analysis. Initially, the titles and abstracts of all articles identified by the search were screened by two researchers independently in line with the inclusion criteria; any disagreements were resolved by consensus. The titles and abstracts of non-quantitative studies and studies that did not apply the health belief model framework to predict vaccination intention were excluded. Full-text articles were obtained for studies whose titles and abstracts met inclusion criteria. All full-text articles were then evaluated to confirm if they reported necessary statistics of HBM constructs–vaccination intention relationships. 

**Figure 1:**
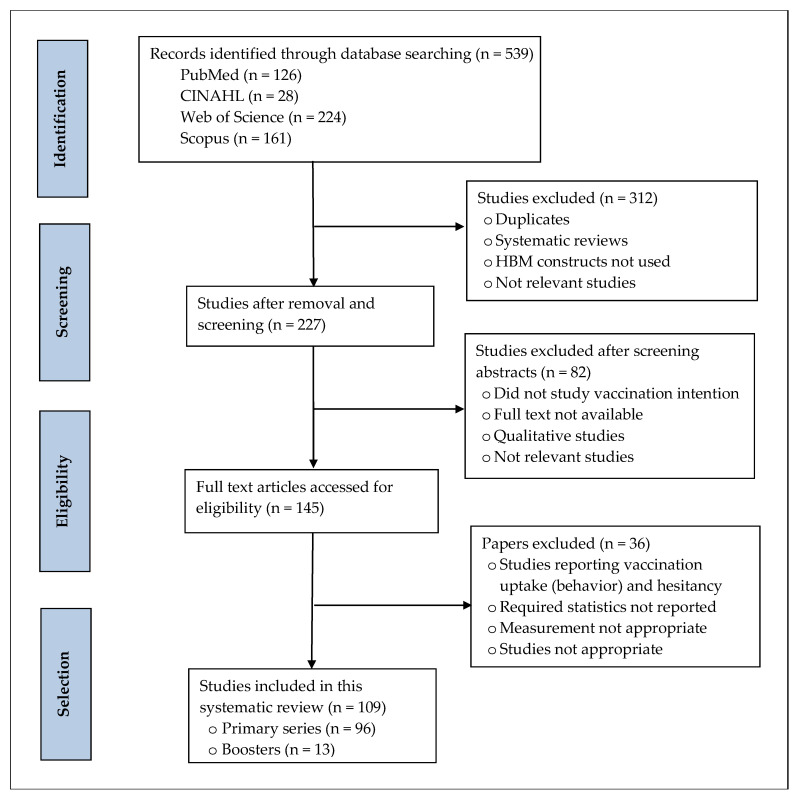
PRISMA flow diagram illustrating literature search.

A PRISMA flow diagram was drawn to demonstrate the study selection process, the number of records identified, screened, and excluded, and the reasons for exclusion (see Figure 1). A total of 539 records were retrieved from the four electronic databases. Of them, 312 records were removed for duplicates, systematic reviews, and studies not using HBM constructs. A total of 82 articles were excluded after screening the abstracts as they were irrelevant or did not study vaccination intention or qualitative studies. The remaining 145 full-text papers were further assessed for eligibility. We included 109 studies that met all inclusion criteria after excluding studies not reporting vaccination intention or acceptance, reporting vaccination uptake (behavior) and hesitancy, not reporting required statistics, or not meeting other criteria.

### 2.3. Data Extraction and Analysis

The same two researchers extracted data from the studies independently. The information extracted consist of the author’s name, data collection year, publication year, study objective, study design, population, sample size, sampling method, measure, statistical analysis technique, the country where the study was conducted, and vaccination rate. We also extracted information on HBM constructs associated with vaccination intention (susceptibility, severity, benefits, barriers, cues to action, self-efficacy, and modifying variables). The outcome variable was COVID-19 vaccination intention. 

Data were analyzed using IBM SPSS Statistics 27. First, the characteristics of studies included in the review were summarized using frequencies and percentages. Next, we reported average vaccination intention rates by country, data collection year, and population. Finally, the prevalence of HBM constructs significantly related to vaccination intention was presented by data collection year, population, and geographic locations (country and continent).

### 2.4. Risk of Bias

To ensure the methodological quality as well as to evaluate the level of bias and to assess specific concerns about potential biases in the database search, selection, data extraction, and synthesis, the ROBIS tool was used as per the guidelines of Whiting et al. [14]. The ratings were used to judge the overall risk of bias. The signaling questions were answered as “yes”, “probably yes”, “probably no”, “no”, or “no information”. The subsequent level of concern about bias associated with each domain was then judged as “low”, “high”, or “unclear”. If the answers to all signaling questions for a domain were “yes” or “probably yes”, the level of concern was judged as low. If any signaling question was answered “no” or “probably no”, then bias exists. 

The same two researchers independently used the ROBIS tool to evaluate risk of bias and to identify studies to be included in the present investigation. Any disagreements were resolved through discussion or a decision made by an expert, a third umpire. Similarly, the selection of databases or digital libraries was also decided with consensus.

## 3. Results

### 3.1. Characteristics of the Included Studies

This systematic review included 109 studies comprising 96 primary series vaccines and 13 booster vaccines. Fifty-seven articles were published in 2022, forty-two in 2021, eight in 2023, and three in 2020 (see Table 1). Thirty-three (33%) were published in *Vaccines*, a peer-reviewed journal, and eight studies appeared in *Human Vaccines & Immunotherapeutics*. Over half of the studies (58/109) collected data in 2021, thirty in 2020, eight in 2022, and eight in 2020–2021. Fifty-nine studies were conducted in Asia, nineteen in North America, fourteen in Europe, and ten in Africa. These studies represent 21 countries, with twenty-one studies from China and eighteen from the USA. 

All studies were cross-sectional in design. The studies included in this review consisted of 174,490 respondents with a sample size ranging from 110 to 18,201 (mean = 1601, SD = 2234.20). Sixty-six articles studied general adult populations, fourteen health care workers, nine parents, and nine college students. Other populations included patients, teachers, employees, and travelers. All studies recruited participants aged 18 years and above. The vast majority of the studies (87.16%) used non-random sampling (convenience sampling); the remaining fourteen used random sampling techniques. Except for two studies that conducted experiments, all other studies collected data using the survey method. Forty-nine studies used SPSS to analyze their data, twenty-three used STATA, and seven used R. Most studies (70%) used regression analysis and ten used structural equation modeling.

### 3.2. Vaccination Intention Rate by Country, Population, and Year

**Figure 2:**
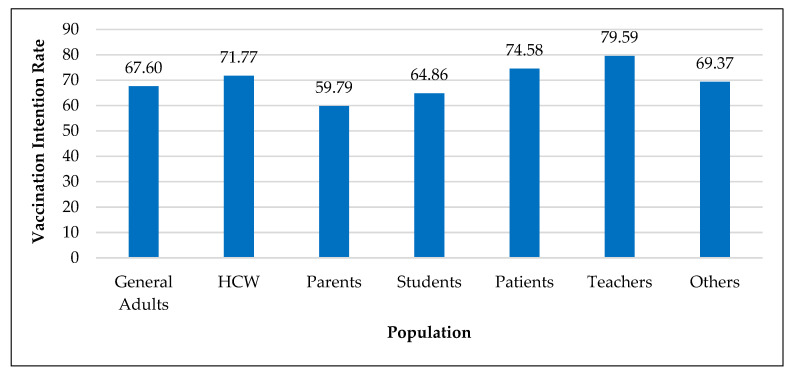
Vaccination intention rate by population.

Overall COVID-19 vaccination intention rate was 68.19% (Std. = 17.58), which ranged from 31% to 97.6%. Average vaccination intention percentages for COVID-19 by country were: Malaysia (94.3%), India (89.3%), Puerto Rico (82.7%), Philippines (77.50%), China (76.34%), Israel (75.72%), UK (74.23%), Vietnam (73.48%), Bangladesh (73.17%), Saudi Arabia (66.18%), USA (65.21%), Ethiopia (55.64%), Sri Lanka (54%), and Romania (46.95%). The overall acceptance rate for the COVID-19 vaccine across all studies increased from 63.68% in 2020 to 70% in 2021 and then remained flat in 2022 (69%). As shown in Figure 2, average vaccination intention rate was highest among teachers (80%), followed by patients (75%), health care workers (72%), and general adults (68%). Only 60% of the parents intended to get their children vaccinated against COVID-19.

### 3.3. HBM Constructs Associated with Vaccination Intention

**Figure 3:**
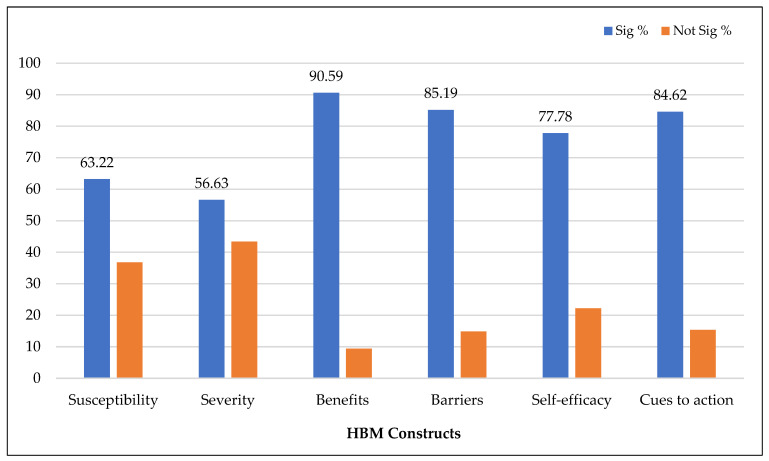
Health belief model constructs predicting primary series vaccination intention.

Table 2 presents the studies that reported significant associations between HBM constructs and COVID-19 vaccination intention. As shown in Figure 3, perceived benefits of COVID-19 vaccination, the most commonly demonstrated HBM construct, predicted vaccination intention in eighty-seven studies (90.59%) for primary series vaccines. Perceived barriers to accepting the vaccine against COVID-19 were found to be inversely associated with vaccination intention in seventy-seven studies (85.19%). Cues to action were found to be positively associated with vaccination intention in fifty-eight studies (84.61%), perceived susceptibility to develop COVID-19 infection in fifty-five studies (63.22%), perceived severity of COVID-19 infection in fifty-one studies (56.63%), and self-efficacy in twenty-nine studies (77.78%). Surprisingly, thirty-six studies (43.37%) reported insignificant associations between perceived severity and vaccination intention. Similarly, over one-third of the studies (36.78%) that examined perceived susceptibility and over one-fifth of the studies (22.22%) that examined self-efficacy were not significant predictors of vaccination intention.

**Figure 4:**
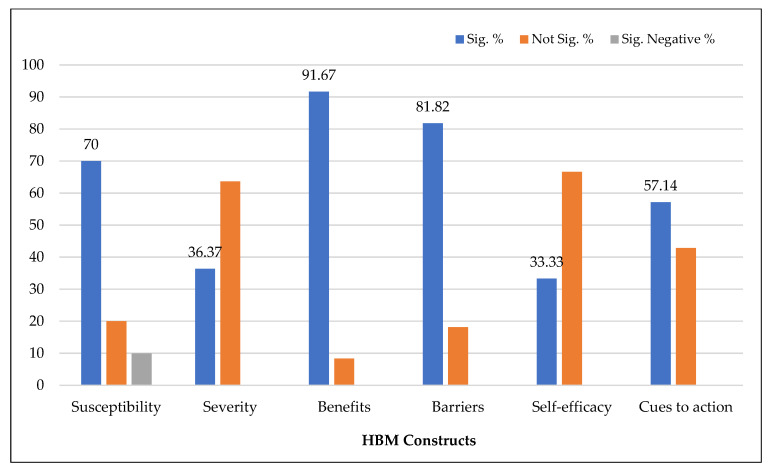
Health belief model constructs predicting booster vaccination intention.

Only thirteen articles used the health belief model to explore the predictors of COVID-19 booster vaccination intention. As presented in Figure 4, perceived benefits, the most commonly demonstrated HBM factor, predicted booster vaccination intention in eleven studies (91.67%). Perceived barriers were negatively related to booster vaccination intention in nine studies (81.82%). Susceptibility was positively associated with booster intention in seven studies (70%). On the contrary, Hu et al. [49] found a negative effect of susceptibility on booster acceptance. Of the eleven studies that examined severity, only four reported perceived severity as a significant determinant of booster intention; however, such an effect was not evident in seven articles (63.64%). In addition, self-efficacy was not significantly associated with booster intention in three out of four studies. Similarly, cues to action did not predict booster intention in three studies (42.86%).

In conclusion, the influence of perceived severity, self-efficacy, and cues to action on vaccination intention declined for boosters. However, the impact of perceived susceptibility slightly increased for boosters.

### 3.4. Modifying HBM Constructs Associated with Vaccination Intention

**Figure 5:**
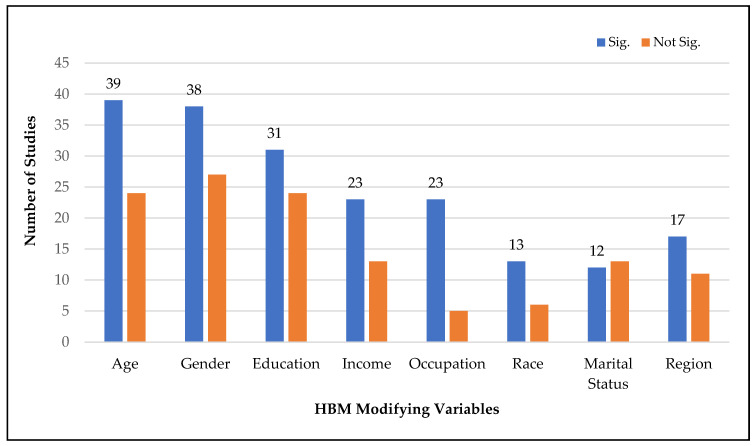
Major HBM modifying variables associated with vaccination intention.

As shown in Figure 5, the most prevalent modifying variable significantly associated with COVID-19 vaccination intention was age (39 studies), followed by gender (38), education (31), income (23), occupation (23), region (17), race (13 studies), and marital status (12). Other frequently explored modifying variables significantly influencing vaccination intention were religion, nationality, political leaning, history of flu or COVID-19 vaccination, history of COVID infection, knowledge of disease or COVID-19, trust in healthcare system, science or media, sources of information, and health status.

### 3.5. HBM Constructs Associated with Vaccination Intention by Data Collection Year, Country, Continent, and Sample

#### 3.5.1. Data Collection Year

**Figure 6:**
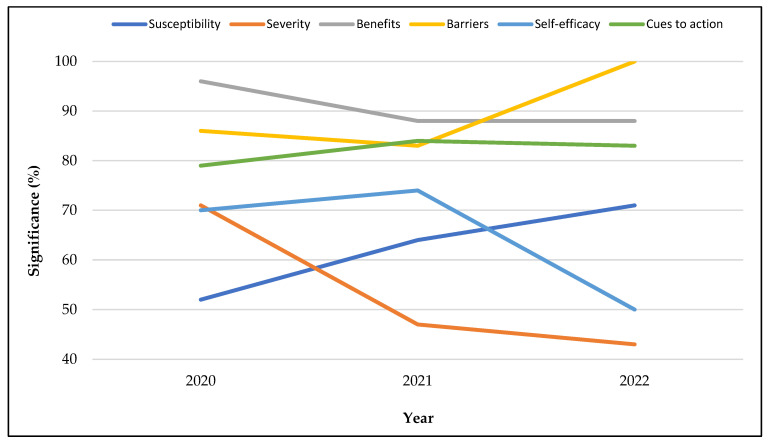
Health belief model constructs associated with vaccination intention by data collection year.

While the effects of perceived susceptibility on vaccination intention increased significantly from 2020 to 2022, the influence of perceived severity declined sharply (see Figure 6). The significant association of perceived barriers with vaccination intention slightly declined from 2020 to 2021, but it skyrocketed in 2022. Conversely, the effect of self-efficacy dipped in 2022. In addition, the role of perceived benefits declined from 2020 to 2021 but remained flat in 2022. Conversely, the influence of cues to action increased in 2021, but slightly declined in 2022.

#### 3.5.2. Geographic Location

**Figure 7:**
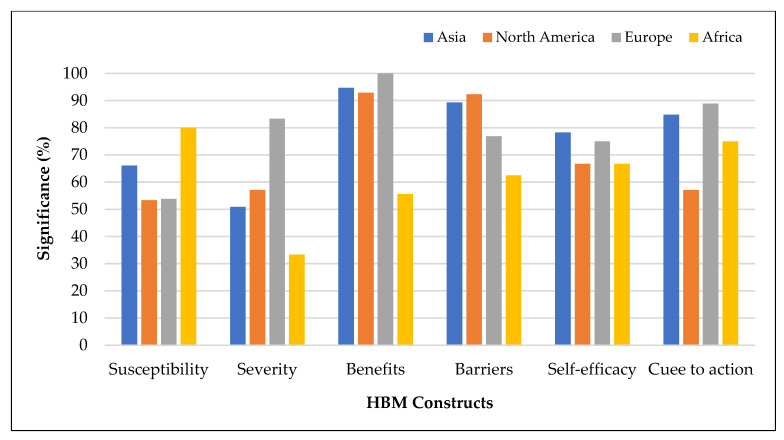
Health belief model constructs associated with vaccination intention by continent with five or more studies.

Figure 7 presents the associations between HBM factors and vaccination intention by continent with five or more studies. All other HBM constructs, except perceived susceptibility, were associated with vaccination intention less frequently in Africa compared to Asia, Europe, and North America. In addition, perceived susceptibility was a less prevalent significant predictor in North America and Europe, compared to Africa and Asia.

**Figure 8:**
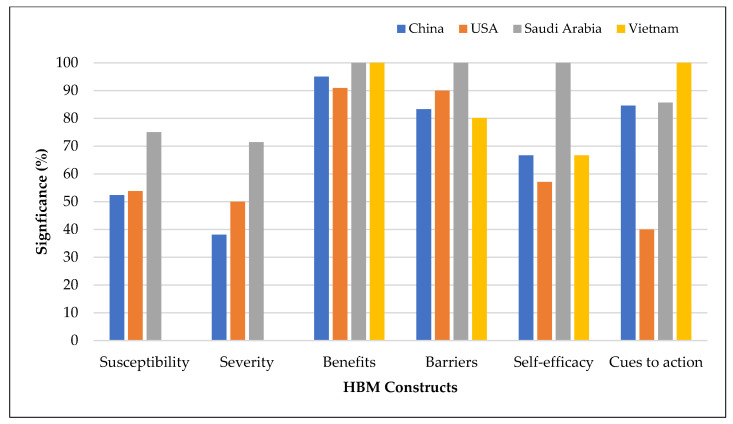
Health belief model constructs associated with vaccination intention by country with five or more studies.

Figure 8 presents the relationships between HBM dimensions and vaccination intention by countries with five or more studies. Perceived susceptibility and perceived severity were more common determinants of vaccination intention in Saudi Arabia than in China and the USA. Perceived severity was the least frequently demonstrated predictor of vaccination intention in China. Self-efficacy and cues to action were less frequently demonstrated predictors in the USA than in China, Saudi Arabia, and Vietnam. Perceived barriers were the more dominant factor influencing vaccination intention in Saudi Arabia than in other countries.

#### 3.5.3. Study Population

**Figure 9:**
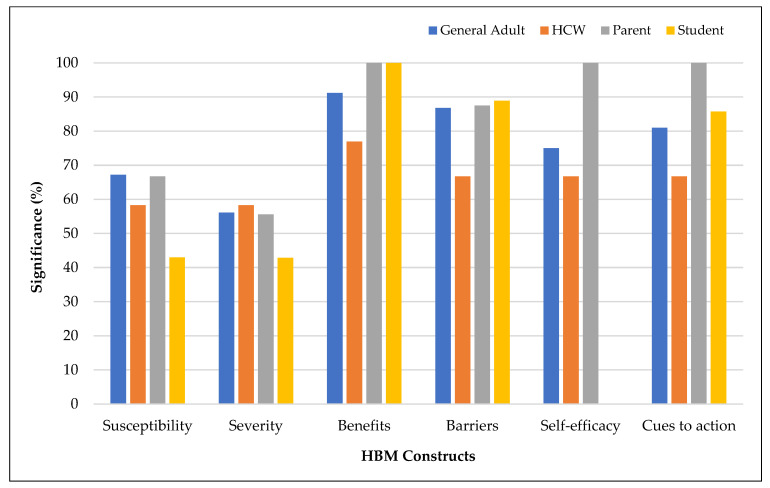
Health belief model constructs associated with vaccination intention by study population.

Figure 9 shows the associations between HBM constructs and vaccination intention by the study population. The effects of perceived susceptibility and perceived severity on vaccination intention were lower among students. Perceived barriers were the least frequently demonstrated predictor among health care workers. Cues to action and self-efficacy had a dominant influence among parents. 

## 4. Discussion and Implications

The results suggest that perceived benefits of receiving the COVID-19 vaccine was the most common HBM construct predicting vaccination intention. Other dominant HBM constructs were perceived barriers to receiving the vaccine and cues to action (i.e., information, people, and events that guided them to be vaccinated). However, perceived susceptibility to developing COVID-19 infection, perceived severity of COVID-19 infection, and perceived self-efficacy of receiving the COVID-19 vaccine were weaker determinants of vaccination intention. The findings of this systematic review provide some support for the health belief model as a useful framework for understanding the facilitators and barriers to COVID-19 vaccination intention. This finding is consistent with previous studies, which suggested similar evidence in the context of influenza vaccination [123,124,125].

Our results indicate that perceived benefits, perceived barriers, and cues to action were the three most frequently demonstrated HBM constructs predicting vaccination intention for both primary series and booster vaccines. Hence, COVID-19 vaccine promotional campaigns should emphasize the benefits of vaccinating against COVID-19. Providing truthful and up-to-date information about the benefits of vaccines can encourage individuals to get vaccinated. Therefore, COVID-19 vaccination communication campaigns may need to progressively shift emphasis from addressing risk perceptions and concerns to stressing the benefits of vaccination for the individual and the community [126].

The results also highlight the importance of identifying the barriers to vaccination (e.g., lack of trust in the government or healthcare system, insufficient knowledge about the benefits of vaccines, misinformation about the coronavirus and vaccines, lack of affordability or shortage of vaccines) and ensuring a course of action to overcome them. In addition, making the vaccine easily accessible (e.g., offering walk-in clinics and mobile vaccination units) can reduce barriers to vaccination and increase uptake. Similarly, offering incentives, such as free or discounted products or services, can motivate individuals to accept vaccines. 

The results also show that increasing the vaccination cue to action is crucial. For example, vaccine recommendations or reminders by trusted authorities, government agencies, public health officials, and healthcare experts can effectively persuade people to accept vaccines. In addition, social media and the social influence of celebrities, politicians, friends, family members, or community leaders can play a crucial role in educating, persuading, and influencing people’s vaccination decisions.

This review reveals that the influence of susceptibility on vaccination intention increased from 2020 to 2022 and was higher for booster doses than for primary series vaccines. On the contrary, severity was a less common predictor of vaccination intention for boosters. These results imply that people were increasingly concerned about being infected by COVID-19. Simultaneously, an increasing number of individuals perceived COVID-19 as a less severe disease. One reason is that the virus might have infected several people after receiving primary series vaccines, and they might have experienced mild systems, similar to traditional viruses such as the common cold and influenza. These counterintuitive beliefs pose a significant obstacle to vaccination. Therefore, the government and other concerned parties promoting vaccines should focus on increasing people’s perceptions of the seriousness of COVID infections.

In conclusion, to combat the ongoing pandemic and to increase vaccine uptake against COVID-19, agencies such as the government, policymakers, and the WHO should take account of health beliefs when designing interventions and public health campaigns encouraging vaccination. However, such initiatives should take into account not only the HBM constructs but also geographic (e.g., country, regions), socioeconomic status (e.g., income, education), and other demographic (e.g., age, gender, and occupation) factors that can influence an individual’s vaccination decision.

## 5. Directions for Future Research

This systematic review synthesized the literature that investigated the relationships between HBM constructs and vaccine intention against COVID-19. However, the findings are mixed. Several studies reported strong correlations between HBM constructs and vaccination intention, but others did not; this is true for both primary series and booster doses. These contradictory results may have been due to several limitations (e.g., research design, study population, data collection approach, measures, analytical approach, and theoretical frameworks) that can be addressed by future studies. 

Our results show that the vast majority of studies that utilized theoretical models were based on HBM and the Theory of Planned Behavior. Hence, future research can examine the applicability of other theories such as the Theory of Reasoned Action, Protection Motivation Theory, Social Cognitive Theory, Self-Determination Theory, Information–Motivation–Behavioral Skills Model, Cognitive Behavioral Theory, Theory of Triadic Influence, Social Network Theory, Diffusion of Innovation Theory, and Social Support Theory. 

All the studies included in this systematic review used cross-sectional data. Thus, future research should apply a longitudinal approach because people’s opinions and health beliefs on COVID-19 and vaccines may change over time [127]. 

Furthermore, this review shows that the vast majority of the studies included in this review used a descriptive/correlational study design and relied on survey methodology. Hence, we recommend causal research and experiments to establish cause-and-effect relationships between the predictors and outcomes. In addition, machine learning techniques and secondary data can be utilized. Finally, qualitative methods such as focus group studies, in-depth interviews, and case studies can provide important insights into vaccine hesitancy and help understand the nuances of vaccination intention across different populations and geographic regions [128].

The studies included in this review used various measures to assess people’s intentions and hesitancy using a slider or a Likert scale. Some of them were dichotomized into vaccine intention and hesitancy. These measures can be misleading and unreliable as they can oversimplify complex attitudes and behaviors related to vaccination against COVID-19. In addition, these measures may not capture the nuances of individuals’ vaccine intentions and hesitancy. Hence, alternative measures (e.g., multiple-item scales that assess different aspects of vaccination attitudes and behaviors) can be developed and used for future research.

A vast majority of studies included in this systematic review used regression analysis, especially logistic regression using dichotomous dependent variables. Thus, we recommend using other statistical analyses (e.g., SEM, linear regression) with a continuous outcome variable. 

Our results indicate that the HBM has been primarily applied to study vaccine intentions of the general adult population, parents, students, and health care workers. Future research should focus on specific and under-represented populations such as deprived communities, ethnic and racial minorities, rural and aged populations, and people with multiple chronic conditions. Likewise, more research is needed to explore understudied regions or countries, especially Australia, Oceania, South America, and African countries. Similarly, comparing high-income versus low-income countries, Western versus non-Western countries, and developed versus developing regions/countries may provide additional insights into the conflicting literature. 

Finally, it is also essential to consider the cultural and social context in which vaccine intention and hesitancy are assessed. Different cultures and social groups may have unique beliefs, values, and experiences related to COVID-19 and vaccination, and these factors can influence attitudes and behaviors towards vaccination. Therefore, it is crucial to use culturally sensitive and culturally appropriate measures when assessing vaccine intention and hesitancy.

## 6. Conclusions

This systematic review synthesizes the findings of quantitative studies examining the associations between HBM constructs and vaccination intention against COVID-19. Our results indicate that perceived benefits, perceived barriers, and cues to action are the most common determinants of vaccination intention. However, perceived susceptibility to developing COVID-19 infection, perceived severity of COVID-19 infection, and perceived self-efficacy of receiving the COVID-19 vaccine were weaker predictors of vaccination intention. In addition, the associations between HBM factors and vaccination intention differed across vaccine type, study year, geographic location, and study population. The results show that the health belief model can be helpful for understanding the facilitators and barriers to COVID-19 vaccination intention.

## Figures and Tables

**Table 1:** Basic characteristics of the studies included in this systematic review.

Author(s)	Year of Publication	Journal	Country	Vaccine Intention %	Population	Sample Size
Al-Hasan et al. [15]	2021	Frontiers in Public Health	NR*	75	General population	372
Al-Metwali et al. [16]	2021	Journal of Evaluation in Clinical Practice	Iraq	62	HCW	1680
Almalki et al. [17]	2022	Frontier in Public Health	Saudi Arabia	38	Parents	4135
Alobaidi [18]	2021	Journal of Multidisciplinary Healthcare	Saudi Arabia	72	General population	1333
Alobaidi and Hashim [19]	2022	Vaccines	Saudi Arabia	71	HCW	2059
Alobaidi et al. [20]	2023	Vaccines	Saudi Arabia	78	Patients	179
An et al. [21]	2021	Health Services Insights	Vietnam	81	Patients	462
Ao et al. [22]	2022	Vaccines	Malawi	61	General population	758
Apuke and Tunca [23]	2022	Journal of Asian and African Studies	Nigeria	55	General population	385
Arabyat et al. [24]	2023	Research in Social and Administrative Pharmacy	Jordan	-	General population	3116
Banik et al. [25]	2021	BMC Infectious Diseases	Bangladesh	66	General population	682
Barattucci et al. [26]	2022	Vaccines	Italy	84	General population	1095
Berg and Lin [27]	2021	Translational Behavioral Medicine	USA	71	General population	350
Berni et al. [28]	2022	Vaccines	Morocco	71	General population	3800
Burke et al. [29]	2021	Vaccine	Australia, Canada, England, New Zealand, USA	73	General population	4303
Cahapay [30]	2022	Journal of Human Behavior in the Social Environment	Philippine	-	Teachers	1070
Caple et al. [31]	2022	PeerJ	Philippines	63	General population	7193
Chu and Liu [32]	2021	Patient Education and Counseling	USA	80	General population	934
Coe et al. [33]	2022	Research in Social and Administrative Pharmacy	USA	63	General population	1047
Duan et al. [34]	2022	Vaccines	China	80	Patients	645
Dziedzic et al. [35]	2022	Frontiers in Public Health	Poland	75	HCW	443
Ellithorpe et al. [36]	2022	Vaccine	USA	60	Parents	682
Enea et al. [37]	2022	Health Communication	Argentina, Australia, Brazil, Canada, Croatia, France, Germany, Greece, Hungary, Italy, Malaysia, Netherlands, Romania, Russia, South Africa, Spain, Turkey, Ukraine, UK, USA	73	General population	6697
Getachew et al. [38]	2022	Frontier in Public Health	Ethiopia	36	HCW	417
Getachew et al. [39]	2023	BMJ Open	Ethiopia	55	Patient	412
Ghazy et al. [40]	2022	IJERPH	EMR	75	General population	2327
Goffe et al. [41]	2021	HVI	UK	62	General population	1660
Goruntla et al. [42]	2022	Asian Pacific Journal of Tropical Medicine	India	89	General population	2451
Guidry et al. [43]	2021	American Journal of Infection Control	USA	60	General population	788
Guidry et al. [44]	2022	International Journal of Environmental Research and Public Health	USA	80	Evangelicals	531
Guillon and Kergall [45]	2021	Public Health	France	31	General population	1146
Handebo et al. [46]	2021	PLOS ONE	Ethiopia	67	Teachers	301
Hawlader et al. [47]	2022	International Journal of Infectious Diseases	Bangladesh, India, Pakistan, Nepal	68	General population	18,201
Hossian et al. [48]	2022	PLOS ONE	Pakistan	73	Students	2865
Hu et al. [49]	2022	Vaccines	China	84	General population	898
Huang et al. [50]	2023	Journal of Environmental and Public Health	China	92	General population	525
Huynh et al. [51]	2022	Asian Pacific Journal of Tropical Medicine	Vietnam	76	HCW	410
Iacob et al. [52]	2021	Frontiers in Psychology	Romania	45	General population	864
Jahanshahi-Amjazi et al. [53]	2022	JEHP	Iran	72	General population	2365
Jiang et al. [54]	2021	HVI	China	72	HCW	1039
Jin et al. [55]	2021	Vaccines	Pakistan		General population	320
Kasting et al. [56]	2022	JMIR Public Health and Surveillance	USA	80	General population	1643
Khalafalla et al. [57]	2022	Vaccines	Saudi Arabia	84	General population	1039
Kabir et al. [58]	2021	Vaccines	Bangladesh	69	General population	697
Lai et al. [59]	2021	Vaccines	China	85	General population	1145
Le An et al. [60]	2021	HVI	Vietnam	77	Students	854
Le et al. [61]	2022	BMC Public Health	Vietnam	58	HCW	911
Lee et al. [62]	2022	JHCPF	Hong Kong	29	General population	800
Li, J.-B. et al. [63]	2022	Vaccine	Hong Kong	-	Parents	11,141
Li, G. et al. [64]	2022	Health Services Research and Managerial Epidemiology	Thailand	67	HCW	226
Liao et al. [65]	2022	Vaccine	Hong Kong	61	General population	4055
Lin et al. [66]	2020	PLOS Neglected Tropical Diseases	China	83	General population	3541
Lin et al. [5]	2021	HVI	China	78	Parents	2026
Liu et al. [67]	2022	IJERPH	China	63	General population	3389
Lopez-Cepero et al. [68]	2021	HVI	Puerto Rico	83	General population	1911
Lyons et al. [69]	2023	Vaccines	Trinidad	60	Patients	272
Mahmud et al. [70]	2021	Vaccines	Saudi Arabia	58	General population	1387
Mahmud et al. [71]	2022	Vaccines	Jordan	84	General population	2307
Maria et al. [72]	2022	Vaccines	Indonesia	89	HCW	1684
Mercadante and Law [73]	2021	Research in Social and Administrative Pharmacy	USA	67	General population	525
Miyachi et al. [74]	2022	Vaccines	Japan	91	Students	1776
Mohammed et al. [75]	2022	Vaccine	Iraq, Jordan, UAE, Oman, Yemen	56	Parents	1154
Morar et al. [76]	2022	IJERPH	Romania	51	General population	110
Nguyen et al. [77]	2021	Risk Management and Healthcare Policy	Vietnam	78	Students	412
Okai and Abekah-Nkrumah [78]	2022	PLOS ONE	Ghana	63	General population	362
Okmi et al. [79]	2022	Cureus	Saudi Arabia	73	General population	1939
Okuyan et al. [80]	2021	International Journal of Clinical Pharmacy	Turkey	75	HCW	961
Otiti-Sengeri et al. [81]	2022	Vaccines	Uganda	98	HCW	300
Patwary et al. [82]	2021	Vaccines	Bangladesh	85	General population	543
Qin et al. [83]	2022a	Vaccines	China	94	General population	3119
Qin et al. [84]	2022b	Frontiers in Public Health	China	88	Parents	1724
Qin et al. [85]	2022c	Frontiers in Public Health	China	83	60 or older	3321
Qin et al. [86]	2023	HVI	China	81	General population	3224
Quinto et al. [87]	2021	Philippine Journal of Health Research and Development	Philippine	93	Teachers	707
Rabin and Durta [88]	2021	Psychology, Health & Medicine	USA	76	General population	186
Reindl and Catma [89]	2022	Expert Review of Pharmacoeconomics & Outcomes Research	USA	66	Parents	30
Reiter et al. [90]	2020	Vaccine	USA	69	General population	2006
Rosental and Shmueli [91]	2021	Vaccines	Israel	82	Students	628
Rountree and Prentice [92]	2022	Irish Journal of Medical Science	Ireland	32	General population	1995
Seangpraw et al. [93]	2022	Frontiers in Medicine	Thailand		General population	1024
Seboka et al. [94]	2021	Risk Management and Healthcare Policy	Ethiopia	65	General population	1160
Shah et al. [95]	2022	Vaccine	Singapore	-	General population	1009
Shmueli [96]	2021	BMC Public Health	Israel	80	General population	398
Shmueli [97]	2022	Vaccines	Israel	65	General population	461
Short et al. [98]	2022	Families, Systems and Health	USA	37	Students	526
Sieverding et al. [99]	2023	Psychology, Health & Medicine	UK and Germany	88	General population	1425
Spinewine et al. [100]	2021	Vaccines	Belgium	58	General population	1132
Ştefănuţ et al. [101]	2021	Frontiers in Psychology	Romania	45	Students	432
Su et al. [102]	2022	Frontiers in Psychology	China	73	General population	557
Suess et al. [103]	2022	Tourism Management	USA	71	Travelers	1478
Tran et al. [104]	2021	Pharmacy Practice	Russia	42	General population	876
Ung et al. [105]	2022	BMC Infectious Diseases	Macao	62	General population	552
Vatcharavongvan et al. [106]	2023	Vaccine	Thailand	90	Parents	1056
Wagner et al. [107]	2022	Vaccines	USA	38	General population	1012
Walker et al. [108]	2021	Vaccines	China	36	Students	330
Wang [109]	2022	Health Communication	China	80	General population	460
Wang et al. [110]	2021	HVI	China	64	Students	833
Wijesinghe et al. [111]	2021	Asia Pacific Journal of Public Health	Sri Lanka	54	General population	895
Wirawan et al. [112]	2022	Vaccines	Indonesia	56	General population	2674
Wong et al. [113]	2020	HVI	Malaysia	94	General population	1159
Xiao et al. [114]	2021	Vaccines	China	56	General population	2528
Yan et al. [115]	2021	Vaccines	Hong Kong	42	General population	1255
Yang et al. [116]	2022	IJERPH	China	82	General population	621
Youssef et al. [117]	2022	PLOS ONE	Lebanon	58	HCW	1800
Yu et al. [118]	2021	HVI	China	72	HCW	2254
Zakeri et al. [119]	2021	Journal of Pharmaceutical Health Services Research	USA	62	Parents	595
Zampetakis and Melas [120]	2021	Appl Psychol Health Well-Being	Greece	44	Employees	1165
Zhang et al. [121]	2023	Vaccines	China	86	General population	1472
Zhelyazkova et al. [122]	2022	Vaccines	Germany	84	HCW	2555

NR* = Country is not reported, but the regions are (North America, the Middle East, Europe, Asia); HCW = Health care workers; IJERPH = International Journal of Environmental Research and Public Health; HVI = Human Vaccines & Immunotherapeutics; JHCPF = INQUIRY: The Journal of Health Care Organization, Provision, and Financing.

**Table 2:** Health belief model constructs significantly associated with vaccination intention.

HBM Construct	Studies
Perceived susceptibility	[17,18,19,22,25,26,28,29,31,33,34,37,38,39,40,42,43,46,47,48,50,57,58,59,62,63,66,67,68,70,71,72,75,78,79,80,81,82,83,84,85,87,88,89,90,91,92,93,94,95,96,98,100,104,105,109,111,113,115,118,120,122]
Perceived severity	[5,15,17,18,22,24,28,29,31,33,34,35,36,38,39,40,41,42,47,48,50,57,58,61,64,65,66,67,68,70,71,74,75,79,80,87,89,90,92,93,97,98,99,100,102,107,113,118,120,121,122]
Perceived benefits	[5,15,16,17,18,19,20,21,22,24,25,28,29,31,32,33,34,35,39,41,42,43,44,45,46,47,48,49,50,51,52,53,54,55,57,58,59,60,61,62,63,66,68,69,70,71,72,73,74,75,76,77,78,79,80,82,83,84,85,86,87,88,89,90,91,96,97,99,100,101,102,103,104,106,108,109,110,111,112,113,114,115,116,117,118,119,120,121,122]
Perceived barriers	[5,15,16,17,18,19,20,21,22,23,25,27,28,29,30,31,32,34,35,39,40,42,43,44,45,46,47,48,49,50,51,52,53,54,55,56,57,58,59,61,62,63,66,68,70,71,73,74,75,77,78,79,80,82,83,85,87,89,91,92,95,98,99,101,102,104,105,106,108,110,112,113,114,115,116,117,119,120,121]
Self-efficacy	[5,17,21,22,28,34,43,53,55,56,57,61,71,75,76,79,82,89,90,92,93,95,96,105,109,114,115,118,121]
Cues to action	[15,16,17,18,20,21,22,23,24,28,29,31,34,42,45,46,47,48,49,51,54,57,58,60,61,62,63,66,68,69,70,71,75,77,79,82,83,85,86,87,89,91,92,93,94,96,97,100,104,106,108,110,113,115,116,117,118,119,122]

## Data Availability

Data generated in this study is available by contacting the first author, Yam B. Limbu, if requested reasonably.
